# Complete microbial genomes for public health in Australia and the Southwest Pacific

**DOI:** 10.1099/mgen.0.000471

**Published:** 2020-11-12

**Authors:** Sarah L. Baines, Anders Gonçalves da Silva, Glen P. Carter, Amy Jennison, Irani Rathnayake, Rikki M. Graham, Vitali Sintchenko, Qinning Wang, Rebecca J. Rockett, Verlaine J. Timms, Elena Martinez, Susan Ballard, Takehiro Tomita, Nicole Isles, Kristy A. Horan, William Pitchers, Timothy P. Stinear, Deborah A. Williamson, Benjamin P. Howden, Torsten Seemann, Communicable Diseases Genomics Network (CDGN)

**Affiliations:** ^1^​ Department of Microbiology and Immunology, The University of Melbourne at the Peter Doherty Institute for Infection and Immunity, Parkville, Victoria 3001, Australia; ^2^​ Microbiological Diagnostic Unit Public Health Laboratory, Department of Microbiology and Immunology, The University of Melbourne at the Peter Doherty Institute for Infection and Immunity, The University of Melbourne, Parkville, Victoria 3001, Australia; ^3^​ Public Health Microbiology, Queensland Reference Centre for Microbial and Public Health Genomics, Forensic and Scientific Services, Queensland Department of Health, Archerfield, Queensland 4108, Australia; ^4^​ Centre for Infectious Diseases and Microbiology – Public Health, Westmead Hospital and NSW Health Pathology, Sydney, New South Wales 2145, Australia; ^5^​ Marie Bashir Institute for Infectious Diseases and Biosecurity, The University of Sydney, Sydney, New South Wales 2006, Australia

**Keywords:** public health microbiology, genomics, reference genomes

## Abstract

Complete genomes of microbial pathogens are essential for the phylogenomic analyses that increasingly underpin core public health laboratory activities. Here, we announce a BioProject (PRJNA556438) dedicated to sharing complete genomes chosen to represent a range of pathogenic bacteria with regional importance to Australia and the Southwest Pacific; enriching the catalogue of globally available complete genomes for public health while providing valuable strains to regional public health microbiology laboratories. In this first step, we present 26 complete high-quality bacterial genomes. Additionally, we describe here a framework for reconstructing complete microbial genomes and highlight some of the challenges and considerations for accurate and reproducible genome reconstruction.

## **Significance as a BioResource to the communit**y

Referenced-based bioinformatic analyses are increasingly being used to enhance public health activities; comparative genomics having been shown to appreciably assist in outbreak investigation and understanding the genetic context underlying clinically relevant phenotypes. However, reference-based analyses are inherently constrained by the genetic similarity of the reference strain to the population being studied and subsequently a catalogue of high-quality reference strains is required to support the diverse analyses undertaken in the public health environment. The genomes reported here represent the first 26 reference strains to be incorporated into a new public health resource; a collection of diverse bacterial pathogens of importance to Australia and the Southwest Pacific (including curated genomic and phenotypic data), that will continue to be added to in a multi-jurisdictional collaboration between public health laboratories in the region. To further support public health activities, we also provide a detailed framework for bacterial genome reconstruction, using a combination of short- and long-read sequence data generated from different platforms. Included is a discussion of the challenges encountered and the considerations made to ensure both accuracy and reproducibility in the construction of these reference genomes.

## Data Summary

All sequencing data and assemblies have been deposited in the National Center for Biotechnology Information (NCBI) database under the BioProject no. PRJNA556438, and are available from figshare: https://doi.org/10.26188/13107509.

Pure cultures of all strains were deposited in the Microbiological Diagnostic Unit Public Health Laboratory (MDUPHL) Reference Culture Collection, and are available on request (mdu-general@unimelb.edu.au).

## Introduction

Whole-genome sequence (WGS) data are now a critical tool in public health microbiology [1–4]. The data can be used to replicate many of the now commonly used microbiological sub-typing methodologies, as well as support epidemiological investigations, such as surveillance and outbreak investigation [5–7]. The appeal of WGS data comes from the promise of a single workflow to process all microbial pathogens, and the provision of easily portable data that promote deeper integration of surveillance and investigation efforts across jurisdictions. This promise is leading to a concerted effort to move microbial public health to a primarily genome-based workflow in numerous countries [8–10], including Australia [11].

Essential to the success of this transition to a genomics workflow is the need to develop catalogues of high-quality complete reference genomes of microbial pathogens [12]. Complete bacterial genomes can provide valuable insights, for instance, into the genomic context of virulence and antimicrobial resistance genes [13], and their possible mechanisms of actions. More importantly, complete genomes are essential for generating accurate phylogenomic analyses, a core requirement of public health surveillance and outbreak response. In this setting, they provide valuable context to identify variable genomic regions across samples in a given study in a computationally efficient manner [14–16].

However, pathogenic bacteria are not generally composed of uniform panmictic populations. Instead, they represent numerous diverse clades, with many being endemic to particular regions or jurisdictions [17–23]. We define the latter as clones that are repeatedly observed in a given region with evidence of ongoing local transmission, but are not commonly observed in other parts of the world; a more practical definition is given by clones observed in local outbreaks for which no suitable reference genome is available in the public domain. This inherent population structure poses a challenge to a successful transition to genomics in public health microbiology laboratories and can significantly reduce the resolution of phylogenomic analyses by influencing the identification of genetic variants [24, 25]. Thus, catalogues of complete genomes will only be effective in supporting a transition to genomics in public health microbiology if they are rich in endemic strains.

Here we present the establishment of a genome catalogue for microbial pathogens of regional importance to Australia and the Southwest Pacific, and describe the first 26 complete genomes to be added. We will continue to build on this resource as further strains are sequenced and assembled.

## Methods

### Whole-genome sequencing

**Fig. 1.**
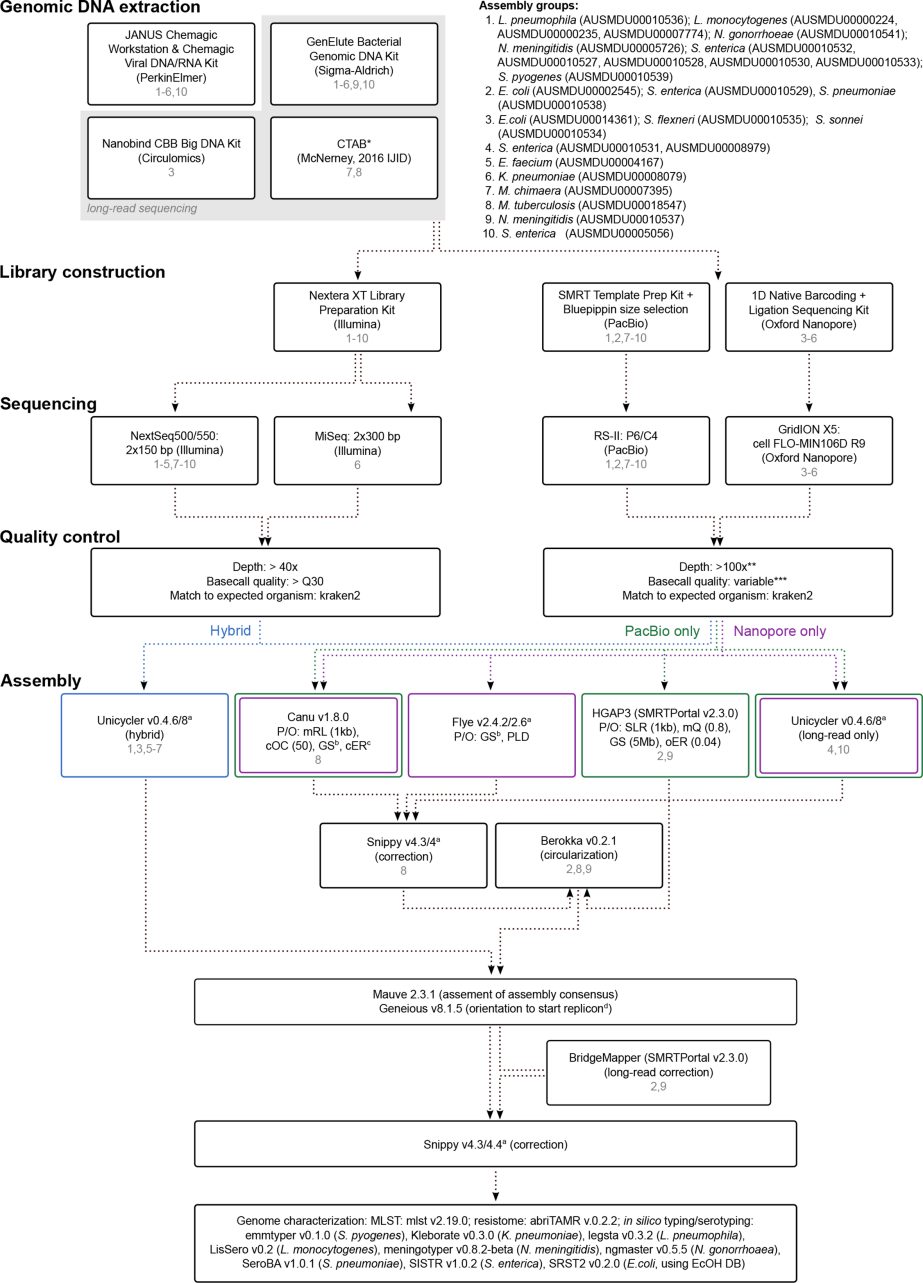
Schematic of the methodology used for sequence data generation and genome assembly. *Modifications to the published CTAB method are decsribed in the methods section. **Nanopore data was filtered to 100x for the expected species size, preferencing quality and length equally using Filtlong v0.2.0; ***PacBio data was filtered using a minimum read quality [mQ] = 0.80; ^a^multiple versions used - refer to the methods section and supplementary tables; P/O = parameters/options (that differ from default); mRL = minimum read length; cOR = corOutCoverage; GS = genome size (^b^set as Mb closest to species average); cER = correctedErrorRate (^c^set as 0.144 for Nanopore or 0.045 for PacBio data); PLD = plasmid flag used; SLR = seed read length; oER = overlapper error rate; ^d^start replicon was *dnaA* for chromosome sequences and *rep* for plasmid sequences, based on prokka annotations.

All strains were grown in appropriate media for the organism following standard laboratory protocols. Whole-genomic DNA was extracted using various methods, selected based on the species to ensure high-quality DNA for short- and long-read sequencing (outlined in Fig. 1). For the Chemagic Viral DNA/RNA kit (PerkinElmer), the GenElute Bacterial Genomic DNA kit (Sigma-Aldrich) and the Nanobind CBB Big DNA kit (Circulomics), extractions were performed as per the manufacturer’s recommendations. For mycobacterial species, the protocol outlined in McNerney *et al*. [26] was used with the following modifications. Growth from five brown and buckle slopes was used for gDNA extraction, dissolved in 500 µl molecular grade (Ultrapure, Life Technologies) water and heated for 60 min at 80 °C. Following incubation with lysozyme (Sigma Aldrich), samples were mixed by manual inversion and all incubation steps were performed at 60 °C. Samples were eluted in EB Buffer (Qiagen, 10 mM Tris/Cl, pH 8.5 buffer) by overnight incubation at 4 °C followed by incubation at 60 °C for 15 min, and then centrifugation.

Short-read genomic DNA was sequenced on either the Illumina NextSeq500/550 (2×150 bp paired-end) or MiSeq (2×300 bp paired-end) platforms. Long-read genomic data were produced on either the PacBio RS-II (P6-C4 chemistry) or Oxford Nanopore GridION X5 (with FLO-MIN106D R9 flow cells) platforms. The DNA extraction, library preparation and sequencing workflow is illustrated in Fig. 1, with strain-specific methodology provided in the figure, Table S1, and the respective NCBI BioSample record (Table 1).

**Table 1.** Demographic and genomic information for the 26 reference genomes

ID	Species	BioSample	Year	Source	MLST (scheme)	Serotype/other type	Sequence ID (replicon type)	Sequence length
AUSMDU00004167	* Enterococcus faecium *	SAMN08628413	2015	Human	ST1421 (efaecium)	–	AUSMDU00004167_01 (Chm) AUSMDU00004167_02 (p01) AUSMDU00004167_03 (p02) AUSMDU00004167_04 (p03) AUSMDU00004167_05 (p04)	2 883 744 bp 206 995 bp 63 221 bp 46 478 bp 6173 bp
AUSMDU00002545	* Escherichia coli *	SAMN13191633	2013	Human	ST11 (ecoli)	O157:H7	AUSMDU00002545_01 (Chm) AUSMDU00002545_02 (p01)	5 553 138 bp 94 640 bp
AUSMDU00014361	* Escherichia coli *	SAMN11008224	2015	Human	ST29 (ecoli)	O26:H11	AUSMDU00014361_01 (Chm) AUSMDU00014361_02 (p01) AUSMDU00014361_03 (p02) AUSMDU00014361_04 (p04)	5 553 138 bp 100 778 bp 100 217 bp 2974 bp
AUSMDU00008079	* Klebsiella pneumoniae *	SAMN07452764	2012	Human	ST258 (kpneumoniae)	KL106:O2v2	AUSMDU00008079_01 (Chm) AUSMDU00008079_02 (p01) AUSMDU00008079_03 (p02) AUSMDU00008079_04 (p03) AUSMDU00008079_05 (p04)	5 449 904 bp 207 351 bp 113 222 bp 43 380 bp 13 841 bp
AUSMDU00010536	* Legionella pneumophila *	SAMN13191634	2016	Human	–	1	AUSMDU00010536_01 (Chm)	3 453 356 bp
AUSMDU00000224	* Listeria monocytogenes *	SAMN13191635	2009	Food	ST122 (lmonocytogenes)	1/2 c,3c:82	AUSMDU00000224_01 (Chm) AUSMDU00000224_02 (p01)	2 931 813 bp 57 553 bp
AUSMDU00000235	* Listeria monocytogenes *	SAMN13191636	2009	Human	ST14 (lmonocytogenes)	1/2a,3a:178	AUSMDU00000235_01 (Chm) AUSMDU00000235_02 (p01)	3 005 026 bp 2776 bp
AUSMDU00007774	* Listeria monocytogenes *	SAMN13191637	2013	Human	ST155 (lmonocytogenes)	1/2a,3a:155	AUSMDU00007774_01 (Chm)	2 964 538 bp
AUSMDU00007395	* Mycobacterium chimaera *	SAMN13191638	2016	Human	ST81 (mycobacteria)	–	AUSMDU00007395_01 (Chm) AUSMDU00007395_02 (p01) AUSMDU00007395_03 (p02) AUSMDU00007395_04 (p03) AUSMDU00007395_05 (p04) AUSMDU00007395_06 (p05)	6 180 270 bp 97 268 bp 39 887 bp 32 137 bp 21 123 bp 13 458 bp
AUSMDU00018547	* Mycobacterium tuberculosis *	SAMN13191639	2017	Human	ST215 (mycobacteria)	–	AUSMDU00018547_01 (Chm)	4 414 769 bp
AUSMDU00010541	* Neisseria gonorrhoeae *	SAMN10920452	2017	Human	ST10899 (neisseria)	1866	AUSMDU00010541_01 (Chm) AUSMDU00010541_02 (p01) AUSMDU00010541_03 (p02)	2 174 817 bp 41 998 bp 4197 bp
AUSMDU00010537	* Neisseria meningitidis *	SAMN13191640	2014	Human	ST11 (neisseria)	w	AUSMDU00010537_01 (Chm)	2 185 677 bp
AUSMDU00005726	* Neisseria meningitidis *	SAMN13191641	2016	Human	ST1655 (neisseria)	Y	AUSMDU00005726_01 (Chm)	2 166 248 bp
AUSMDU00010532	* Salmonella enterica* subsp. *enterica* serovar Birkenhead	SAMN13191642	2015	Human	ST424 (senterica)	Birkenhead	AUSMDU00010532_01 (Chm) AUSMDU00010532_02 (p01)	4 692 229 bp 329 074 bp
AUSMDU00010527	* Salmonella enterica* subsp. *enterica* serovar Enteritidis	SAMN13191643	2017	Human	ST3304 (senterica)	Enteritidis	AUSMDU00010527_01 (Chm) AUSMDU00010527_02 (p01)	4 642 207 bp 41 464 bp
AUSMDU00010528	* Salmonella enterica* subsp. *enterica* serovar Enteritidis	SAMN13191644	2017	Human	ST1972 (senterica)	Enteritidis	AUSMDU00010528_01 (Chm)	4 703 625 bp
AUSMDU00005056	* Salmonella enterica* subsp. *enterica* serovar Hvittingfoss	SAMN05589873	2016	Human	ST434 (senterica)	Hvittingfoss	AUSMDU00005056_01 (Chm)	4 744 949 bp
AUSMDU00010531	* Salmonella enterica* subsp. *enterica* serovar Saintpaul	SAMN13191645	2016	Human	ST50 (senterica)	Saintpaul	AUSMDU00010531_01 (Chm)	4 731 476 bp
AUSMDU00008979	* Salmonella enterica* subsp. *enterica* serovar Typhimurium	SAMN13191646	2012	Human	ST34 (senterica)	I 4 [5], 12:i:-	AUSMDU00008979_01 (Chm) AUSMDU00008979_02 (p01)	5 022 086 bp 144 821 bp
AUSMDU00010529	* Salmonella enterica* subsp. *enterica* serovar Typhimurium	SAMN13191647	2015	Human	ST19 (senterica)	I 4 [5], 12:i:-	AUSMDU00010529_01 (Chm) AUSMDU00010529_02 (p01) AUSMDU00010529_03 (p02)	4 865 665 bp 93 865 bp 93 769 bp
AUSMDU00010530	* Salmonella enterica* subsp. *enterica* serovar Typhimurium	SAMN13191648	2017	Human	ST34 (senterica)	I 4 [5], 12:i:-	AUSMDU00010530_01 (Chm) AUSMDU00010530_02 (p01)	4 964 749 bp 4251 bp
AUSMDU00010533	* Salmonella enterica* subsp. *enterica* serovar Virchow	SAMN13191649	2016	Human	ST16 (senterica)	Virchow	AUSMDU00010533_01 (Chm) AUSMDU00010533_02 (p01)	4 705 038 bp 3691 bp
AUSMDU00010535	* Shigella flexneri *	SAMN13191650	2017	Human	ST245 (ecoli)	2a	AUSMDU00010535_01 (Chm) AUSMDU00010535_02 (p01) AUSMDU00010535_03 (p02) AUSMDU00010535_04 (p03)	4 723 195 bp 234 182 bp 83 548 bp 4692 bp
AUSMDU00010534	* Shigella sonnei *	SAMN13191651	2017	Human	ST152 (ecoli)	g	AUSMDU00010534_01 (Chm) AUSMDU00010534_02 (p01) AUSMDU00010534_03 (p02) AUSMDU00010534_04 (p03) AUSMDU00010534_05 (p04) AUSMDU00010534_06 (p05) AUSMDU00010534_07 (p06) AUSMDU00010534_08 (p07) AUSMDU00010534_09 (p08)	4 837 733 bp 108 107 bp 80 987 bp 57 073 bp 6774 bp 5219 bp 5114 bp 3715 bp 2690 bp
AUSMDU00010538	* Streptococcus pneumoniae *	SAMN13191652	2014	Human	ST199 (spneumoniae)	19A	AUSMDU00010538_01 (Chm)	2 090 744 bp
AUSMDU00010539	* Streptococcus pyogenes *	SAMN13191653	2013	Human	ST101 (spyogenes)	Emm89	AUSMDU00010539_01 (Chm)	1 746 807 bp

### Genome assembly

Before *de novo* assembly, all sequence data underwent quality control (QC) to ensure sufficient depth and basecall quality, and that the sequence data represented the expected organism based on a kraken2 [27] analysis (Fig. 1). Confirmation of *Shigella sonnei* identification was performed by phylogenetic analysis of the strains against samples described elsewhere [28]. In the case of PacBio data, provided that the above QC requirements were met, the consensus circular subreads fastq files were concatenated and used as the input for *de novo* assembly. In the case of Nanopore data, sequence data were basecalled onboard the GridION X5 using ONT’s Guppy basecaller v3.2.4 with the high accuracy protocol. Demultiplexing and adaptor trimming were performed using Porechop v0.2.4 (https://github.com/rrwick/Porechop). Default parameters were used with two exceptions: to be kept, a read required (i) both barcodes to be identified with (ii) a minimum identity of 85 %. Long-read datasets were then filtered using Filtlong v0.2.0 (https://github.com/rrwick/Filtlong), to a final dataset equivalent to 100-fold coverage for the expected genome size, weighting read length and quality equally.

All genomes were assembled using four different approaches. Three were applied to all datasets, one for PacBio data only, and one for ONT data only, as outlined in Fig. 1.

Hybrid assembly with Unicycler v0.4.6 or v0.4.8 [29]. All default parameters were used, providing both the short- and long-read sequence data.
Long read-only assembly with Unicycler v0.4.6 or v0.4.8 [29]. All default parameters were used, providing only long-read sequence data. Following assembly, contigs underwent a single round of error correction with the short-read sequence data using Snippy v4.3 or v4.4 (https://github.com/tseemann/snippy).
Long read-only assembly with Canu v1.8.0 [30]. Default parameters were used with the following exceptions: (i) *genomeSize* was set as ‘3m’ for small genomes (e.g. *Listeria *, *Streptococcus *, *Enterococcus *) or ‘6m’ for large genomes (e.g. *Mycobacteria*, *Salmonella *, *Escherichia *). (ii) the *correctedErrorRate* was set as ‘0.045’ for PacBio data or ‘0.144’ for Nanopore data. (iii) *minReadLength* was set as ‘1000’ and (iv) *corOutCoverage* as ‘50’. If highly fragmented, a second assembly was performed lowering this parameter to ‘20’. Following assembly, contigs underwent a single round of error correction with the short-read sequence data using Snippy v4.3 or v4.4, and then circularization was attempted using berokka v0.2.1 (https://github.com/tseemann/berokka).
(PacBio) Long read-only assembly with HGAP3, SMRTPortal v2.3.0 [31]. Default parameters were used with the following exceptions: (i) *seed read length* was set to ‘1 kb’, (ii) *minimum read quality* was set to ‘0.80’, (iii) *genome size* was set to ‘5000000’ for all assemblies (based on in-house testing) and (iv) the *over-lapper error rate* was set to ‘0.04’. Following assembly, circularization was attempted using berokka v0.2.1.
(ONT) Long read-only assembly with Flye v2.4.2 or v2.6 [32]. Default parameters were used with the following exceptions: (i) *genome-size* was set as ‘3m’ (for small genomes) or ‘6m’ (for large genomes), and both --*meta* and --*plasmid* flags were used to improve plasmid recovery [33]. Following assembly, contigs underwent a single round of error correction with the short-read sequence data using Snippy v4.3 or v4.4, and then circularization was attempted using berokka v0.2.1.

Following assembly, all draft genomes were compared for structural consistency and a single assembly was selected. Features considered during comparison were: (i) inter-assembly variation in genome size and consistency with expected size for the species; (ii) number and location of ribosomal RNA gene; (iii) broad structural similarity as assessed visually from an alignment generated with Mauve v1.1.1 [34], looking for large differences (i.e. >5 kb) including inversions, duplications and deletions; (iv) representation of small replicons (e.g. plasmids and other mobile elements). When selecting a final assembly, the hybrid assembly approach was prioritized. If required, selection between long read-only assembly approaches was based on which produced a structure that most closely represented the consensus of the assembly outputs. In general, this was HGAP3 (PacBio) or Flye (ONT), followed by Unicycler, then Canu; an order that is consistent with a benchmarking study that established performance standards for these assemblers in the context of long-read genome assembly [33].

Following selection, the final assembly was assessed for orientation to an appropriate start replicon and adjusted if required. The assembly then underwent a final error correction with the short-read sequence data using Snippy v4.3 or v4.4, run in an iterative manner until no variants were detected. This was also performed on hybrid assemblies generated by Unicycler, even though the software performs its own short-read correction, the reasoning for which is explained below. Strain-specific assembly information is illustrated in Fig. 1, and provided in Table S1 and the respective NCBI BioSample record (Table 1).

Sequences representing plasmids were additionally checked for similarity to published sequences deposited in the NCBI database (https://www.ncbi.nlm.nih.gov/). Of the 39 plasmids recovered, only one was identified as novel; that belonging to the *Salmonella enterica* subsp. *enterica* serovar Birkenhead AUSMDU00010532. This sequence contained genes consistent with plasmid replication machinery.

### Genome characterization

*In silico* multi-locus sequence typing (MLST) was performed using mlst v2.19.0 (https://github.com/tseemann/mlst), employing the pubMLST schemes [35]. Antimicrobial resistance genes were detected using abriTAMR v0.2.2 (https://github.com/MDU-PHL/abritamr), and outputs were filtered with minimum gene length and minimum nucleotide identify cut-offs of 90 %. *In silico* typing/serotyping was performed using emmtyper v0.1.0 (https://github.com/MDU-PHL/emmtyper) for *S. pyogenes *, Kleborate v0.3.0 (https://github.com/katholt/Kleborate) for *Klebsiella pneumoniae *, legsta v0.3.2 (https://github.com/tseemann/legsta) for *Legionella pneumophila *, LisSero v0.2 (https://github.com/MDU-PHL/LisSero) for *Listeria monocytogenes *, meningotype v0.8.2-beta (https://github.com/MDU-PHL/meningotype) for *Neisseria meningitidis *, ngmaster v0.5.5 (https://github.com/MDU-PHL/ngmaster) for *Neisseria gonorrhoeae *, SeroBA v1.0.1 [36] for *Streptococcus pneumoniae *, SISTR v1.0.2 [37] for *Salmonella enterica *, and SRST2 v0.2.0 [38] using the EcOH DB for *Escherichia coli *.

## Results and Discussion

Here, we present 26 high-quality complete bacterial reference genomes. Strains were selected to represent a broad range of organisms of importance for public health in Australia and the Southwest Pacific. These included foodborne (e.g. *L. monocytogenes *, and *S. enterica *), waterborne (e.g. *L. pneumophila *), sexually transmitted (e.g. *N. gonorrhoeae *) and other pathogens of public health importance (e.g. *K. pneumoniae *, *Mycobacterium* sp., *N. meningitidis *). In some cases, we chose the strains because of their relevance to local surveillance and outbreak requirements as well as their virulence or antimicrobial resistance (AMR) phenotypes (e.g. colistin-resistant *S. enterica *, carbapenem-resistant *K. pneumoniae* and vancomycin-resistant *Enterococcus faecium *). Presented in Table 1 is a summary of the demographic and genomic characteristics (including *in silico* MLST and serotypes) of the 26 reference genomes. Phenotypic antimicrobial susceptibility data (when testing is appropriate for the given species) and the matched genotypic AMR profiles are presented in Table 2.

**Table 2.** Phenotypic antimicrobial susceptibility data and resistance genes profiles for the 26 reference genomes

ID	Species	Phenotypic antimicrobial susceptibility			Genotypic resistance	Genomic location
		Susceptible	Resistant	Method		
AUSMDU00004167	* E. faecium *	LZD	AMP; PEN; TEC; VAN	Vitek2	aac(6′)-I; ant(9)-Ia; dfrG; eat(A); erm(A); msr(C) aac(6′)-Ie - aph(3′)-IIIa aph(3′)-IIIa; erm(B); vanA	AUSMDU00004167_01 (Chm) AUSMDU00004167_02 (p01) AUSMDU00004167_04 (p03)
AUSMDU00002545	* E. coli *	AMK; AMC; AMP; CFZ; FEP; CTX; FOX; CAZ; CIP; GEN; MEM; NIT; NOR; TZP; TIM; TOB; TMP; SXT		Vitek2	–	
AUSMDU00014361	* E. coli *	AMK; AMC; AMP; CFZ; FEP; CTX; FOX; CAZ; CIP; GEN; MEM; NIT; NOR; TZP; TIM; TOB; TMP; SXT		Vitek2	–	
AUSMDU00008079	* K. pneumoniae *		AMK; AMC; AMP; CFZ; FEP; FOX; CAZ; CRO; CIP; GEN; MER; NOR; TZP; TIM; TOB; TMP	Vitek2	blaSHV-11; fosA aadA2; aph(3′)-Ia; catA1; dfrA12; mph(A); sul1 blaKPC-2; blaOXA*; blaTEM-1 blaSHV-12 aac(6′)-Ib	AUSMDU00008079_01 (Chm) AUSMDU00008079_02 (p01) AUSMDU00008079_03 (p02) AUSMDU00008079_04 (p03) AUSMDU00008079_05 (p04)
AUSMDU00010536	* L. pneumophila *			np	–	
AUSMDU00000224	* L. monocytogenes *			np	–	
AUSMDU00000235	* L. monocytogenes *			np	–	
AUSMDU00007774	* L. monocytogenes *			np	–	
AUSMDU00007395	* M. chimaera *			np	–	
AUSMDU00018547	* M. tuberculosis *	INH; RIF; EMB; PZA		BACTEC MGIT 960	–	
AUSMDU00010541	* N. gonorrhoeae *	CRO	PEN; CIP; TET HL-AZM†	Agar dilution	tet(M)	AUSMDU00010541_02 (p01)
AUSMDU00010537	* N. meningitidis *	CRO; CIP; RIF	PEN	Agar dilution	–	
AUSMDU00005726	* N. meningitidis *	PEN; CRO; CIP; RIF		Agar dilution	–	
AUSMDU00010532	* S. enterica* subsp. *enterica* serovar Birkenhead	AMC; AMP; FEP; CTX; CAZ; CIP‡; MEM; NIT; TZP; TIM; TOB; TMP; SXT		Agar dilution	fosA7	AUSMDU00010532_01 (Chm)
AUSMDU00010527	* S. enterica* subsp. *enterica* serovar Enteritidis	AMC; AMP; FEP; CTX; CAZ; CIP‡; MEM; NIT; TZP; TIM; TOB; TMP; SXT		Agar dilution	–	
AUSMDU00010528	* S. enterica* subsp. *enterica* serovar Enteritidis	AMC; AMP; FEP; CTX; CAZ; CIP‡; MEM; NIT; TZP; TIM; TOB; TMP; SXT		Agar dilution	–	
AUSMDU00005056	* S. enterica* subsp. *enterica* serovar Hvittingfoss	AMC; AMP; FEP; CTX; CAZ; CIP‡; MEM; NIT; TZP; TIM; TOB; TMP; SXT		Agar dilution	–	
AUSMDU00010531	* S. enterica* subsp. *enterica* serovar Saintpaul	AMC; AMP; FEP; CTX; CAZ; CIP‡; MEM; NIT; TZP; TIM; TOB; TMP; SXT		Agar dilution	–	
AUSMDU00008979	* S. enterica* subsp. *enterica* serovar Typhimurium	AMC; AMP; FEP; CTX; CAZ; CIP‡; MEM; NIT; TZP; TIM; TOB; TMP; SXT	GEN§	Agar dilution	aac(3)-IId; aph(3′′)-Ib; aph(6)-Id; mcr-3.1; qnrS1; sul2; tet(A)	AUSMDU00008979_02 (p01)
AUSMDU00010529	* S. enterica* subsp. *enterica* serovar Typhimurium	AMC; AMP; FEP; CTX; CAZ; CIP‡; MEM; NIT; TZP; TIM; TOB; TMP; SXT		Agar dilution	–	
AUSMDU00010530	* S. enterica* subsp. *enterica* serovar Typhimurium	AMC; FEP; CTX; CAZ; CIP‡; MEM; NIT; TZP; TOB; TMP; SXT	AMP; TIM (I);	Agar dilution	aph(3′′)-Ib; aph(6)-Id; blaTEM-1; sul2; tet(B)	AUSMDU00010530_01 (Chm)
AUSMDU00010533	* S. enterica* subsp. *enterica* serovar Virchow	AMC; AMP; FEP; CAZ; CRO; CIP‡; MEM; NIT; TZP; TIM; TOB; TMP; SXT		Agar dilution	–	
AUSMDU00010535	* S. flexneri *	FEP; CTX; CAZ; CIP‡; MEM; NIT; TZP; TOB	AMC; AMP; TIM (I); TMP; SXT	Agar dilution	aadA1; blaEC; blaOXA-1; catA1; tet(B) aadA5; blaTEM-1; dfrA17; erm(B); mph(A); sul1	AUSMDU00010535_01 (Chm) AUSMDU00010535_03 (p02)
AUSMDU00010534	* S. sonnei *	FEP; CTX; CAZ; CIP‡; MEM; NIT; TZP; TOB	AMC; AMP; TIM; TMP; SXT	Agar dilution	aadA1; blaEC; dfrA1; sat2 aadA5; blaTEM-1; dfrA17; erm(B); mph(A); sul1	AUSMDU00010535_01 (Chm) AUSMDU00010535_03 (P02)
AUSMDU00010538	* S. pneumoniae *	PEN||; CTX; CRO||; CLI; ERY||; LVX; LZD; TET; SXT; VAN		Vitek2	–	
AUSMDU00010539	* S. pyogenes *	PEN||; CTX; CRO; CLI; ERY||; LVX; LZD; TET||; SXT; VAN||		Vitek2	–	

*blaOXA identified in AUSMDU00008079 has an internal stop codon; gene functionality is unknown.

†HL-AZM: high level azithromycin resistance; MIC >256 mg/L in this isolate.

‡Ciprofloxacin MICs for *Salmonella* and *Shigella* species was not tested below 0.25 mg/L; all isolates tested as <0.25 mg/L.

§Isolate demonstrated *in vitro* resistance to gentamicin, MIC=16 mg/L.

||Phenotypic susceptibility to antibiotics indicated were confirmed by Etest.

Amikacin, AMK; amoxicillin/clavulanic acid, AMC; ampicillin, AMP; azithromycin, AZM; benzylpenicillin, PEN; cefazolin, CFZ; cefepime, FEP; cefotaxime, CTX; cefoxitin, FOX; ceftazidime, CAZ; ceftriaxone, CRO; ciprofloxacin, CIP; clindamycin, CLI; ethambutol, EMB; erythromycin, ERY; gentamicin, GEN; isoniazid, INH; levofloxacin, LVX; linezolid, LZD; meropenem, MEM; nitrofurantoin, NIT; norfloxacin, NOR; piperacillin/tazobactam, TZP; pyrazinamide, PZA; rifampicin, RIF; teicoplanin, TEC; tetracycline, TET; ticarcillin/clavulanic acid, TIM; tobramycin, TOB; trimethoprim, TMP; trimethoprim/sulfamethoxazole, SXT; vancomycin, VAN; np, phenotypic testing was not performed.

With the increasing use of genomics in public health investigation, high-quality reference genomes have become a key resource but one that must be continually updated with shifts in circulating microbial lineages, the emergence of new outbreak clones and the ongoing spread of genetic material encoding clinically relevant phenotypes. The advances in long-read sequencing technology have made it increasingly cost-effective to generate the data needed to construct reference genomes. However, missing are pipelines to automate the downstream assembly process. Such a pipeline would need to be capable of generating accurate and reproducible genomes and reliably handle genetically diverse datasets with minimal manual intervention. Following is a discussion about our experiences with reconstructing the 26 genomes described in this paper and considerations that should be made in the generation of such a bioinformatic pipeline.

### Assembly approaches; one size does not fit all

Of the 104 assemblies performed (4 per genome), only 54 were designated as ‘complete’ when considered in isolation; defined as an assembly output that included (i) a chromosomal contig that was circular and of an appropriate size for the species, and (ii) if present, circular plasmid contig(s) that matched published sequences (based on a nucleotide alignment and length comparison), or in the case of AUSMDU00010532 (* Salmonella* Birkenhead) carried genes encoding known plasmid replication machinery.

The remaining 50 assemblies were designated as either ‘draft’ (*n*=14) – contig(s) represented a full-length chromosome and plasmid(s), when present, but were not circularized (examination of the contig ends identified a sequence overlap, indicating that the entire replicon was reconstructed) – or ‘failed’ (*n*=36), with most representing fragmented assemblies. There were only two assembly attempts in which the assembly approach produced no output (Table S2, available in the online version of this article).

Overall, the hybrid assembly approach produced the highest number of ‘complete’ assemblies (19/26). However, every approach produced an assembly designated as ‘failed’ on at least three datasets. This indicated that there is no one-size-fits-all approach to reconstructing the genomes of a diverse collection of strains, consistent with the performance of the various assemblers reported by Wick and Holt [33]. Subsequently, a pipeline developed for long-read genome assembly in public health would need to incorporate multiple assembly tools and approaches to maximize performance.

### Structural consistency; assembling the same dataset twice does not always give the same result

With the examination of a few assembly metrics it is reasonably straightforward to detect large errors in reconstructed genomes. For example, fragmented or linear contigs, significant length inconsistencies for a given species, and the absence of genes of interest. However, there are a range of more subtle errors that may go undetected when an assembly output is considered in isolation. These include structural rearrangements (inversions, duplications and deletions), absence of plasmids and other small replicons, and the presence of superfluous contigs that do not represent true replicons. All of which could contribute to error in a reference-based analysis.

Therefore, all assemblies (with the exception of those that were highly fragmented or for which no output was produced) were compared for structural consistency (as outlined in the methods). This identified 28 assemblies with structural inconsistencies (Table S2), 22 of which were initially deemed ‘complete’ or ‘draft’ (due to linear contigs) when considered in isolation. Of these, 10 were inconsistent due to the absence of small replicon(s) and 16 due to the presence of inversions, duplications or deletions (the affected regions ranging in the 10s to 100s of kb). Four assemblies contained both inconsistencies.

These findings highlight a significant issue with reproducibility. Every assembly approach generated at least one output that was deemed to be structurally inconsistent. We will not comment on which assemblies were ‘correct’ or which approach produced the least number of ‘incorrect’ assemblies, as we have made our judgments based on which genome structure was most commonly observed in the assembly outputs, and have not preformed the required laboratory-based experiments to determine which is biologically correct.

Regardless, our experience highlights that to achieve complete and reproducible genome reconstruction, multiple assembly approaches should be used, and their outputs compared. This creates challenges for pipeline generation, with the comparison of assembly outputs still requiring some level of manual curation. For example, of the 68 assemblies classified as ‘complete’ or ‘draft’ (ignoring structural inconsistencies), the outputs of 20 contained small superfluous contigs; artefacts of the assembly approaches that were not representative of true replicons (Table S2). A small number of these were circularized due to repetitive sequence and without comparison and curation would have been included in the final assembly.

Ultimately, which assembly is selected as the ‘final’ one is dependent on the species, available sequence data and the assembly approach(es) used. Our method was to select the assembly output that most closely represented the consensus of all outputs, was biologically consistent with the species and contained the characteristics expected for the strain (i.e. AMR determinants), favouring the hybrid assembly output when it met these requirements.

Since the submission of our manuscript, our conclusions have been additionally supported by observations from others (R. Wick, personal communication), leading to the recent release of TryCycler (https://github.com/rrwick/Trycycler), a tool that may help automate comparisons of multiple genome assemblies and the identification of a consensus assembly. We look forward to contributing to the development of TryCycler by comparing our manually curated outputs with those produced by the tool.

### Nanopore vs PacBio, and are short-read sequence data really needed?

There was no clear difference in the assembly outputs in terms of genome completeness or structural inconsistencies between the PacBio and ONT datasets. The main distinction between the two approaches is that the latter is more cost-effective for bacterial genomes and requires smaller amounts of input DNA. It has become standard practice at MDUPHL to undertake short-read sequencing on the same genomic DNA that is used for long-read sequencing. Our findings from these assemblies indicate that it is worthwhile as the hybrid assembly approaches, which utilizes both datasets in the assembly process, generated the highest number of ‘complete’ genomes (Table S2). Both short- and long-read sequence data can be generated in similar time frames in public health laboratories. However, multiplexing enhances cost savings and outside of urgent public health activities, there is often a delay in the generation of long-read data, waiting until a sufficient number of samples are available to maximize the output of the platform.

Another common use for short-read sequence data in this context is in genome polishing; a process of correction by mapping the short reads to the long-read assembly and identifying the consensus. Multiple iterations of long-read polishing can result in 99.9% consensus between the reads and the assembly. However, in a bacterial genome this still equates to thousands of potential errors, the majority of which are insertions or deletions due to the known issue with basecalling homopolymers during long-read sequencing.

We recommend short-read polishing because it provides relatively cheap, high-depth coverage across the genome and helps overcome the higher error rate in long-read sequence data, further reducing the number of potential errors in the final genome, a feature that is very important for the mapping-based analyses conducted by public health laboratories to investigate transmission. For consistency, we opted to short-read polish all final assemblies, including the hybrid assemblies generated with Unicycler, which undertakes a short-read correction step but utilizes a different tool from that used in our laboratory for mapping-based analysis.

### Orientation; to start at *dnaA* or not

While it is a preference with minimal to no impact on downstream analysis, we support the practice of orientating contigs to start at genes encoding the replication machinery. This is straightforward for chromosomes, only requiring the identification of *dnaA*. It is more challenging for plasmids, which carry diverse and often multiple replication-associated genes, with the identification of which is responsible for replication not always simple. Of the assembly tools used, only Unicycler included a step to orientate contigs. Of the 21 final assemblies constructed by either the hybrid or long read-only approaches using Unicycler, the chromosome orientation was correct in 16, with the other 5 requiring adjustment (Table S2).

### Next steps: pipeline generation and open access

With long-read sequencing on a trajectory to become part of routine public health practice, the next required step is the development of a pipeline to automate the process of assembly. As indicated, such a pipeline would need to incorporate multiple tools and approaches, compare outputs for inconsistencies, handle data from different platforms and from diverse species, and preferably orientate final contigs to a suitable start replicon. As mentioned, TryCycler appears to be a promising step in this direction. Outside of this, such a pipeline should (i) be amenable to rapidly changing sequencing technologies. (ii) Be designed in a way to enable to development of genus- and/or species-specific workflows (e.g. through specifying organism-specific parameters through a config file that could be easily shared). In this scenario, we would envision such configuration parameters being tuned on large datasets, such as those housed at the NCBI Pathogen Portal, and shared with the wider community. It has not been mentioned, but assembly challenges were noted for specific species due to genome structural complexity, and repetitive and/or recombinant regions. (iii) Most importantly, such a pipeline would need to be proven to reliably and reproducibility generate data that are consistent with both short-read sequence and phenotypic data to be accredited for use in a public health laboratory. Here again, we would advocate taking advantage of the large numbers of assembled genomes already on the NCBI Pathogen Portal, as well as the ATCC’s high-quality reference genome collection (https://www.atcc.org/en/Documents/Marketing_Literature/Genome_Portal_Technical_Document.aspx).

Another important step is open access for newly generated reference genomes. While there are a number of challenges to uploading public health data (including legal, ethical and computational considerations), where possible reference genomes and linked phenotypic and demographic data should be openly available to maximize the use of these public health resources. We have chosen to focus first on building our reference dataset with predominantly endemic clones. However, a clone that is endemic in one region may be an imported clone in another, and open access to reference genome collections will enable other laboratories to access high-quality genomes for their own public health activities. To further improve this resource, additional genomic characteristics could be included, such as characterization of key mobile genetic elements or genomic regions that may interfere with reference-based analyses (e.g. integrated phage); information that may help public health laboratories with reference selection and use.

## Conclusions

We report the establishment of a new public health resource; a collection of high-quality reference genomes and matched phenotypic data to support regional public health activities. We will continue to add to this resource as part of a multi-jurisdictional collaboration. Additionally, we share our process for genome reconstruction, highlighting some of the challenges and considerations for accurate, reproducible and eventually automated assembly of high-quality complete microbial genomes of medical and public health relevance.

## Supplementary Data

Supplementary material 1
